# Supplementary material for: A multi-functional nine channels full-spectrum light emitting diode color temperature palette
Source: PLoS One. 2025 Aug 22;20(8):e0327723. doi: 10.1371/journal.pone.0327723 (PMC12373186; doi:10.1371/journal.pone.0327723)
Supplement: S1 — (DOCX) [file pone.0327723.s001.docx]

The code and data used in the article can be accessed through this link：**https://doi.org/10.5281/zenodo.15517037**
