# Supplementary material for: A multi-functional nine channels full-spectrum light emitting diode color temperature palette
Source: PLoS One. 2025 Aug 22;20(8):e0327723. doi: 10.1371/journal.pone.0327723 (PMC12373186; doi:10.1371/journal.pone.0327723)
Supplement: Attachment — Submitted filename: Response to Reviewers.docx [file pone.0327723.s002.docx]

Dear Editor,

We thank the reviewer for the constructive comments.

In the revised version, we have made some changes in line with the reviewer's remarks. A summary of the changes and our response to the reviewer's comments is appended below.

1. The manuscript relies solely on MATLAB-based simulation. No experimental or hardware-based demonstration is presented to validate the proposed model or assess real-world implementation challenges such as thermal management, control electronics, or spectrum stability. The inclusion of a prototype or even preliminary experimental data would greatly strengthen the manuscript.

Response: The focus of this article is on the construction of a theoretical model and its verification using simulations. The aim is to fully explore, optimize, and verify the core principles and algorithms of the proposed model through MATLAB simulations, thereby ensuring the validity and rationality of the theoretical model. This is helpful in reducing the trial-and-error cost and improves the efficiency during the subsequent experimental stage. In this paper, we have demonstrated certain results at the theoretical simulation level and completed the optimization of key parameters and the verification of the algorithm's feasibility. Currently, we are working on the hardware construction. We plan to build the corresponding experimental platform to test the theoretical model through practical tests and further improve the system.

2. Although the authors briefly mention other multi-channel approaches, they do not provide a direct quantitative comparison. Including a comparative table summarizing key metrics (Ra, Rf, Rg, Duv, MELR, CAF) versing 4-, 5-, or 6-channel designs would clarify the specific advantages and performance enhancements of the nine-channel configuration.

Response: To address the comments of the reviewer, in the revised version, we have added Table 3 and a detailed discussion. As shown in Table 3, the key parameters of the nine-channel LED color temperature palette developed in this study are compared with those of four-channel [21], five-channel [25], and six-channel LEDs [22]. The average adjustment accuracy of the correlated color temperature (CCT) is indicated in parentheses following the CCT adjustment range in the table. A dash ("–") denotes that the parameter was not addressed in the respective study or scenario.

The four-, five-, and six-channel LEDs demonstrated relatively low modulation accuracy in CCT adjustment, whereas the nine-channel palette achieved high precision, with an accuracy of 1 K. In terms of color rendering, the palette consistently outperformed the other configurations, delivering a higher Color Rendering Index (CRI) across all scenarios. The use of the TM-30 standard (Rf, Rg) further confirmed that the palette met high color rendering requirements. Additionally, the palette exhibited a lower Duv value compared to the other LEDs, indicating superior chromaticity. Regarding non-visual effects, the palette achieved a lower Circadian Action Factor (CAF) in low-CCT scenarios, suggesting reduced blue light stimulation—making it particularly suitable for relaxation environments. Furthermore, the Melanopic Equivalent Daylight (MELR) performance of the nine-channel palette met the essential requirements previously discussed.

Table 3. Comparison of key parameters of four-channel, five-channel, six-channel and nine-channel LEDs

|  | Four-channel LED | Five-channel LED | Six-channel LED | Nine-channel LED |
| --- | --- | --- | --- | --- |
| CCT(K) | Product-showcase: 2971-6568(513K)  Office-at-night: 3018-6608(513K)  Bedroom-at-night: 2991-6670(525K) | 5:30am-12:03pm:3638-5737  (150K)  12:30pm-19:04pm:3554-5694  (151K) | Work: 3552-6633(616K)  Leisure: 1783-3921(428K)  Daytime indoor:  2792-5655(477K) | Night fall: 2700-3300(1K)  Morning/afternoon:  3300-5300(1K)  Midday: 5300-6500(1K) |
| Ra | Product-showcase: 95  Office-at-night: 81  Bedroom-at-night: 89 | 5:30am-12:03pm: 93  12:30pm-19:04pm: 92 | Work: 85  Leisure: 84  Daytime indoor:97 | Night fall: 97  Morning/afternoon: 97  Midday: 97 |
| Duv | \|Duv\|< 0.005 | \|Duv\|< 0.004 | \|Duv\|< 0.0039 | \|Duv\|< 0.001 |
| R_g_ | - | - | - | 96 |
| R_f_ | - | - | - | 101 |
| MELR(mw/lm) | - | - | - | Night fall: 0.6-0.82  Morning/afternoon: 0.67-1.05  Midday: 0.829-1.14 |
| CAF(blm/lm) | Product-showcase: 0.426-0.887  Office-at-night: 0.453-0.934  Bedroom-at-night: 0.410-0.881 | 5:30am-12:03pm: 0.548-0.837  12:30pm-19:04pm: 0.525-0.834 | Work: 0.530-1.022  Leisure: 0.097-0.471  Daytime indoor:- | Night fall: 0.289-0.429  Morning/afternoon: 0.334-0.586  Midday: 0.450-0.656 |

3. The LED spectra are modeled using ideal Gaussian distributions. This simplification may not reflect the asymmetrical and secondary peaks seen in real LEDs, particularly phosphor-converted devices. The authors should discuss the limitations of this assumption and its impact on simulation of fidelity.

Response: To address the comments of the reviewer, in the revised version, we have added Table 4 and a detailed discussion. In developing the multi-channel LED model, an ideal Gaussian distribution function was initially employed to simulate the spectral power distribution (SPD) of monochromatic LEDs. However, this approach does not account for the inherent asymmetry observed in real LEDs, potentially limiting the applicability of the simulation results in practical scenarios. To address this, an asymmetry factor was introduced into the Gaussian distribution function to evaluate its impact on simulation outcomes.

The asymmetric Gaussian distribution was constructed by dividing the symmetric function into left and right halves at the central wavelength, introducing a 5% difference in area between the two sides. In Table 4, the first column (Group 1) represents a case where the left-half area is 5% larger than the right, while the second column (Group 2) shows the opposite, with the right-half area 5% larger than the left. The third column serves as the control group, representing the ideal symmetric model. The results indicate that a color temperature modulation accuracy of 1 K can still be maintained even with the introduction of asymmetry. In terms of color rendering, both Ra and Rf remained unchanged in Groups 1 and 2. Rg remained stable in Group 1 but showed a slight decrease of approximately 1% in Group 2 compared to the control group. For non-visual parameters, the absolute values of the boundary value change ratios ranged from a minimum of 0.7% to a maximum of 6.53%.

The following is the detailed calculation process (not included in the manuscript): When constructing the asymmetric Gaussian distribution function, we divide the function into left and right halves with the central wavelength as the dividing line, and reflect the asymmetry according to the different areas of the two sides. Here, we set the area difference between the two sides to be 5%. The first column in the table shows that the area of the left part of the Gaussian distribution function is 5% more than that of the right part (Group 1), the second column shows that the area of the right part of the Gaussian distribution function is 5% more than that of the left part (Group 2), and the third column is the ideal Gaussian distribution model (control group). It can be seen from Table 4 that when the Gaussian distribution function is asymmetric, the color temperature can still achieve a modulation accuracy of 1K. In terms of color rendering, the Ra and Rf indicators remain unchanged; the Rg indicator remains unchanged in Group 1 and decreases by about -1% in Group 2 compared with the control group. Among the non-visual effect indicators, we compare the change ratios of the minimum and maximum boundary values.

Group 1:

Night fall period: The change ratio of the upper boundary of MELR is 4.76%, and the change ratio of the lower boundary is 1.83%.

Morning/afternoon period: The change ratio of the upper boundary of MELR is 0.95%, and the change ratio of the lower boundary is -3.58%.

Midday period: The change ratio of the upper boundary of MELR is 2.63%, and the change ratio of the lower boundary is 2.05%.

Similarly:

Night fall period: The change ratio of the upper boundary of CAF is 6.53%, and the change ratio of the lower boundary is 2.77%.

Morning/afternoon period: The change ratio of the upper boundary of CAF is 6.14%, and the change ratio of the lower boundary is 2.69%.

Midday period: The change ratio of the upper boundary of CAF is 3.66%, and the change ratio of the lower boundary is 2.44%.

Group 2:

Night fall period: The change ratio of the upper boundary of MELR is 0.85%, and the change ratio of the lower boundary is -4.67%.

Morning/afternoon period: The change ratio of the upper boundary of MELR is -2.86%, and the change ratio of the lower boundary is -5.97%.

Midday period: The change ratio of the upper boundary of MELR is -1.75%, and the change ratio of the lower boundary is -4.22%.

Similarly:

Night fall period: The change ratio of the upper boundary of CAF is 0.7%, and the change ratio of the lower boundary is -5.19%.

Morning/afternoon period: The change ratio of the upper boundary of CAF is 1.02%, and the change ratio of the lower boundary is -3.89%.

Midday period: The change ratio of the upper boundary of CAF is -1.68%, and the change ratio of the lower boundary is -4.67%.

Table 4. The key parameter values after adjusting the symmetry of the Gaussian distribution function.

|  | Area on left side 5% larger than that on the right side | Area on right side 5% larger than that on the left side | Symmetrical |
| --- | --- | --- | --- |
| CCT(K) | Night fall: 2700-3300(1K)  Morning/afternoon: 3300-5300(1K)  Midday: 5300-6500(1K) | Night fall: 2700-3300(1K)  Morning/afternoon: 3300-5300(1K)  Midday: 5300-6500(1K) | Night fall:2700-3300(1K)  Morning/afternoon:3300-5300(1K)  Midday:5300-6500(1K) |
| Ra | 97 | 97 | 97 |
| Duv | \|Duv\|< 0.001 | \|Duv\|< 0.001 | \|Duv\|< 0.001 |
| R_g_ | 101 | 100 | 101 |
| R_f_ | 96 | 96 | 96 |
| MELR(mw/lm) | Night fall: 0.611-0.859;  Morning/afternoon: 0.646-1.06  Midday: 0.846-1.17 | Night fall: 0.572-0.827  Morning/afternoon: 0.630-1.02  Midday: 0.794-1.12 | Night fall: 0.6-0.82  Morning/afternoon: 0.67-1.05  Midday: 0.829-1.14 |
| CAF(blm/lm) | Night fall: 0.297-0.457;  Morning/afternoon: 0.343-0.622  Midday: 0.461-0.680 | Night fall: 0.274-0.432;  Morning/afternoon: 0.321-0.592  Midday: 0.429-0.645 | Night fall: 0.289-0.429  Morning/afternoon: 0.334-0.586  Midday: 0.450-0.656 |

4. There is no evaluation of how robust the simulation results are to variations in LED flux ratios or FWHM values. A sensitivity analysis on these parameters would provide insights into the stability of the optimized performance under practical variations.

Response: To address the comments of the reviewer, in the revised version, we have added Table 5 and a detailed discussion. Also, we have analyzed the impact of changes in the FWHM of LEDs on the simulation results. This analysis contributes to a deeper understanding of the stability and robustness of the nine-channel LED color temperature palette in practical applications.

Table 5 presents the influence on key parameters when the FWHM of the LEDs is adjusted by ±10%. The third column (Control Group) represents the baseline FWHM values used in this study. The first column (Group 1) corresponds to a scenario where the FWHM is reduced by 5% relative to the control group, while the second column (Group 2) reflects a 5% increase. As shown in Table 5, a color temperature modulation accuracy of 1 K is maintained even when the FWHM is varied. In terms of color rendering, both Ra and Rf values remained unchanged in Groups 1 and 2 compared to the control group. The Rg value remained stable in Group 1 but decreased by approximately 1% in Group 2. For non-visual parameters, the absolute values of the boundary value change ratios ranged from a minimum of 0.72% to a maximum of 12.29%.

The following is the detailed calculation process(not included in the manuscript): The third column of the table shows the full width at half maximum (FWHM) in the original text, serving as the control group. The first column indicates that the LED's FWHM is 5% smaller than that of the control group, and the second column shows that the LED's FWHM is 5% larger than that of the control group. As can be seen from Table 1, when the FWHM of the lamp bead is adjusted by ±10%, the color temperature can still reach a modulation accuracy of 1K. Regarding color rendering, compared with the control group, the Ra and Rf values of the first and second groups remain unchanged. The Rg value of the first group remains the same, and the Rg value of the second group decreases by about 1%. For the non-visual effect indicators, we compared the percentage changes of the minimum and maximum boundary values.

Group 1:

During the Night Fall period, the percentage change of the upper boundary of MELR is -1.34%, and that of the lower boundary is -6%.

During the morning/afternoon period, the percentage change of the upper boundary of MELR is -2.86%, and that of the lower boundary is -5.97%.

During the midday period, the percentage change of the upper boundary of MELR is -2.63%, and that of the lower boundary is -4.34%.

Similarly, during the Night Fall period, the percentage change of the upper boundary of CAF is -3.49%, and that of the lower boundary is -8.65%.

During the morning/afternoon period, the percentage change of the upper boundary of CAF is -4.44%, and that of the lower boundary is -8.68%.

During the midday period, the percentage change of the upper boundary of CAF is -5.34%, and that of the lower boundary is -6.67%.

Group 2:

During the Night Fall period, the percentage change of the upper boundary of MELR is 6.34%, and that of the lower boundary is 2.5%.

During the morning/afternoon period, the percentage change of the upper boundary of MELR is 7.62%, and that of the lower boundary is 2.54%.

During the midday period, the percentage change of the upper boundary of MELR is 3.51%, and that of the lower boundary is 0.72%.

Similarly, during the Night Fall period, the percentage change of the upper boundary of CAF is 3.49%, and that of the lower boundary is 7.96%.

During the morning/afternoon period, the percentage change of the upper boundary of CAF is 12.29%, and that of the lower boundary is 5.69%.

During the midday period, the percentage change of the upper boundary of CAF is 6.55%, and that of the lower boundary is 2.67%.

Table 5. The key parameter values after adjusting the FWHM of the LEDs by ±10%.

|  | Lower 10% FWHM | Higher 10% FWHM | Original FWHM |
| --- | --- | --- | --- |
| CCT(K) | Night fall: 2700-3300(1K)  Morning/afternoon: 3300-5300(1K)  Midday: 5300-6500(1K) | Night fall: 2700-3300(1K)  Morning/afternoon: 3300-5300(1K)  Midday: 5300-6500(1K) | Night fall: 2700-3300(1K)  Morning/afternoon: 3300-5300(1K)  Midday: 5300-6500(1K) |
| Ra | 97 | 97 | 97 |
| Duv | \|Duv\|< 0.001 | \|Duv\|< 0.001 | \|Duv\|< 0.001 |
| R_g_ | 101 | 100 | 101 |
| R_f_ | 96 | 96 | 96 |
| MELR(mw/lm) | Night fall: 0.564-0.809  Morning/afternoon: 0.630-1.02  Midday: 0.793-1.11 | Night fall: 0.615-0.872  Morning/afternoon: 0.687-1.13  Midday: 0.835-1.18 | Night fall: 0.6-0.82  Morning/afternoon: 0.67-1.05  Midday: 0.829-1.14 |
| CAF(blm/lm) | Night fall: 0.264-0.414  Morning/afternoon: 0.305-0.560  Midday: 0.420-0.621 | Night fall: 0.312-0.444  Morning/afternoon: 0.353-0.658  Midday: 0.462-0.699 | Night fall: 0.289-0.429  Morning/afternoon: 0.334-0.586  Midday: 0.450-0.656 |

5. The manuscript would benefit from language polishing. There are grammatical errors, awkward phrasing, and redundancy throughout the text. For example, phrases such as "serve as a color temperature palette" and "the values...are attained" should be revised for correctness and clarity.

Response: We have polished the language of the article.

6. Several figures lack sufficient clarity, proper labeling, and detailed captions. Figures 4 through 8, in particular, need improved resolution and annotations to aid reader comprehension. Axis labels, units, legends, and figure captions should be made more informative.

Response: The labels of each figure have been corrected to correspond with the figure captions. To improve the clarity, the captions of Figures 4, 5, 8, and 9 have been rewritten, and Figures 4 and 6 have been properly annotated. Regarding the image clarity, the images were clear when I uploaded them to the system, but they became unclear after the platform integrated them into a PDF file. However, they are clear again after downloading.

7. Define all abbreviations at first use and maintain consistency (e.g., SPD, MELR, CAF).

Response: The abbreviations have been defined when they are used for the first time and consistency has been maintained.

8. Include discussion on control hardware and how 1K CCT tuning precision is practically achieved.

Response: To address the comments of the reviewer, in the revised version, we have added Figure 3 and a detailed discussion. In the overall hardware design, the driving circuit consists of a terminal control module, a non-isolated step-down module, a drive control module, a constant-current drive module, and a light source module. The connections between the modules are shown in Fig 3. The terminal control module converts the luminous fluxes of the nine LEDs corresponding to the CCTs from 2700K to 6500K into duty cycles and transmits them to the drive control module. The non-isolated step-down module steps down the external 48V power supply to the 3.3V voltage required by the drive control module through a DC-DC step-down chip. The drive control module reads the duty-cycle data from the terminal control module and converts it into PWM(Pulse Width Modulation) signals through a single-chip microcomputer. The constant-current drive module connects the PWM signals output by the single-chip microcomputer to the constant-current drive chip. The constant-current drive chip then outputs currents in different proportions according to different duty cycles. The light source module is composed of the nine LED matrices in Table 1 connected in series or in parallel.

Fig 3. Hardware design.

9. Reformat equations for clarity, ensuring all symbols and parameters are defined within the text.

Response: The formulas have been reformatted, and it has been ensured that all the symbols and parameters are defined in the text.

10. To enhance the scholarly depth of the manuscript and align with recent advancements in tunable and circadian LED systems, the authors are strongly encouraged to cite the following relevant studies: OSA Continuum, 2019, Vol. 2, Issue 8, pp. 2413–2427. DOI: 10.1364/OSAC.2.002413 and Near-Unity PLQY of Cs₃Cu₂X₅ (X = Cl, Br) for High-Efficiency White Light-Emitting Diodes with Exceptional Color Quality, Advanced Materials, 2025, Article No. 2500083. DOI: 10.1002/adma.202500083. These references will support the discussion of tunable spectrum design and reinforce the context for high-quality full-spectrum LED development.

Response: In the revised version, we have cited these two articles. The section of the manuscript where these papers are cited is as follows:

“It is worth mentioning that the current exploration of metal halide perovskite materials[9] and the attempt to obtain white light by combining the five different emission lights of organolead halide perovskites[10] have also added rich research perspectives to this field.”

The funders had no role in study design, data collection and analysis, decision to publish, or preparation of the manuscript.

We look forward to hearing from you.

Sincerely yours,

Hui Cao

On behalf of all authors
